# Discovery of Novel Allopurinol Derivatives with Anticancer Activity and Attenuated Xanthine Oxidase Inhibition

**DOI:** 10.3390/molecules21060771

**Published:** 2016-06-20

**Authors:** Yong Li, Ting-Ting Cao, Shanchun Guo, Qiu Zhong, Cai-Hu Li, Ying Li, Lin Dong, Shilong Zheng, Guangdi Wang, Shu-Fan Yin

**Affiliations:** 1 College of Chemistry, Sichuan University, Chengdu, Sichuan 610064, China; liyong562@126.com (Y.L.); Caotingting987@163.com (T.-T.C.); licaihu@yeah.net (C.-H.L.); scdxliying@163.com (Y.L.); donglilhy@163.com (L.D.); 2 RCMI Cancer Research Center, Xavier University of Louisiana, New Orleans, LA 70125, USA; sguo@xula.edu (S.G.); qzhong@xula.edu (Q.Z.); szheng@xula.edu (S.Z.); 3 Department of Chemistry, Xavier University of Louisiana, New Orleans, LA 70125, USA

**Keywords:** allopurinol derivatives, cytotoxicity, human hepatoma carcinoma cell, XOD inhibition

## Abstract

A series of pyrazolo[3,4-d]pyrimidine derivatives related to allopurinol has been synthesized and evaluated for its cytotoxicity against a panel of three cancer cell lines as well as its xanthine oxidase (XOD) inhibitory activities. Among them, compound **4** showed potent cytotoxicity with IC_50_ values of 25.5 and 35.2 μM against human hepatoma carcinoma cell lines, BEL-7402 and SMMC-7221, respectively. The anticancer activity of **4** was comparable to that of Tanespimycin (17-*N*-allylamino-17-demethoxy geldanamycin, 17-AAG) that inhibited the growth of BEL-7402 and SMMC-7221 cells at IC_50_ values of 12.4 and 9.85 μM, respectively. However, unlike allopurinol, which is also a strong inhibitor of XOD, compound **4** is a much weaker XOD inhibitor, suggesting that the anticancer activities of the allopurinol derivatives may not be associated with XOD inhibition. Moreover, the cytotoxicity of **4** toward normal cells is significantly lower than that of 17-AAG, making **4** a promising lead compound for further optimization of structure-activity relationships that may lead to anticancer agents of clinical utility.

## 1. Introduction

**Figure 1:**
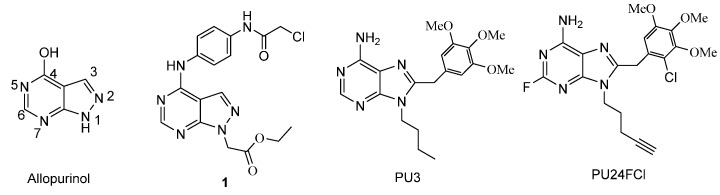
Structures of allopurinol, compound **1**, PU3, and PU24FCl.

Allopurinol (4-hydroxyl-1*H*-pyrazolo[3,4-d]pyrimidine, Figure 1), a prototypical xanthine oxidase (XOD) inhibitor, has been used to treat hyperuricemia and its complications including chronic gout for several decades [1]. Yasuda *et al.* reported that allopurinol exerts cytotoxity on human hormone-refractory prostate cancer cells in combination with tumor necrosis factor-related apoptosis-inducing ligand [2]. Many other pyrazolo[3,4-d]pyrimidine derivatives [3] were extensively explored for their potential in the treatment of cancer by targeting casein kinase 1 (CK1) [4], hepatocyte growth factor receptor (c-MET) [5], erythropoietin-producing human hepatocellular receptors B (EphB) [6], vascular endothelial growth factor receptor 2 (VEGFR2) [6,7,8,9,10], Fms-like tyrosine kinase 3 (FLT3) [7,11], rearranged during transfection proto-oncogene (RET) [8,10], epidermal growth factor receptor (EGFR) [10], MARK (mitogen-activated protein kinases) signaling [9] and Wnt (Wingless-related integration site )/β-catenin signaling [11].

Purine-based cancer therapeutics are well known in clinical applications [12]. Distinct from the traditional cytotoxic drugs, new types of purine analogs as anti-tumor drugs have been designed and developed to target the Hsp 90 protein involved in driving the cancer phenotype [13,14,15,16,17]. PU3 (Figure 1) is a lead compound for this type of small-molecule Hsp 90 inhibitor of purine analogs [13]. Optimization of PU3 led to PU24FCl, which exhibited wide-ranging anti-cancer activities at similar doses in all tested tumor types by specifically and potently inhibiting Hsp 90, while normal cells were 10- to 50-fold more resistant to these effects [14,18]. The successful design and development of PU3 and its simple analogs as anti-tumor agents suggest that further structural modifications to allopurinol might lead to novel analogs with improved pharmacological properties.

**Scheme 1:**
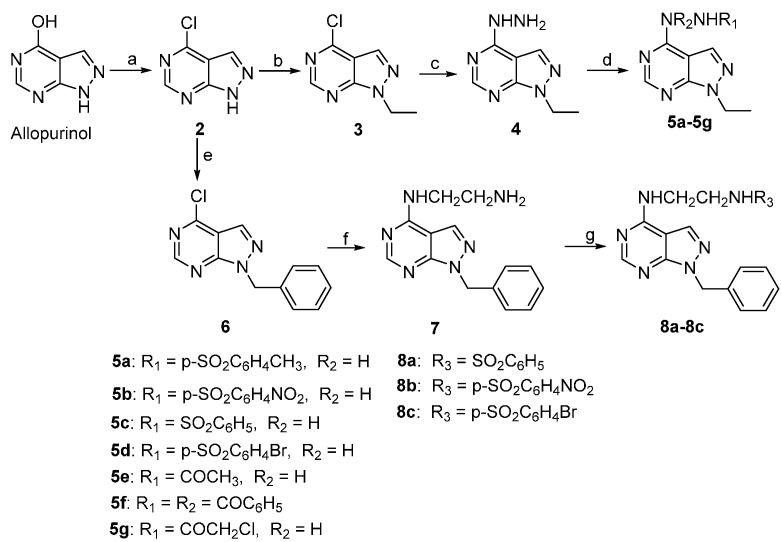
Synthetic route of allopurinol derivatives. *Reagents and conditions*: (**a**) POCl_3_, (CH_3_)_2_NC_6_H_5_, 80 °C; (**b**) BrCH_2_CH_3_, triethylamine (TEA), KI, DMF, rt; (**c**) H_2_NNH_2_.H_2_O, CH_3_CN, rt; (**d**) RSO_2_Cl or RCOCl, pyridine, THF, 0 °C or TEA, CH_2_Cl_2_, 0 °C; (**e**) BrCH_2_C_6_H_5_, TEA, KI, DMF, rt; (**f**) H_2_NCH_2_CH_2_NH_2_, CH_3_CN, rt; and (**g**) RSO_2_Cl, TEA, DMF, 0 °C.

In a previous study, we chose allopurinol as a lead compound and synthesized a panel of pyrazolo[3,4-d]pyrimidine derivatives in the hope that some of the analogs may possess desirable anticancer properties [19]. However, only compound **1** of these derivatives showed potency against human hepatoma carcinoma cells 7402 and 7221 comparable to Tanespimycin (17-AAG), which is a heat shock protein 90 (Hsp 90) inhibitor and is currently in Phase II clinical trials [20]. It is also of note that, while the alkylating moiety present on the C-4 substituent of **1** may contribute to its potency, introduction of a carboxylic acid moiety as the N-1 substitution group appeared to abolish the activity entirely [19,21]. Thus, the dramatic loss of activity in all other allopurinol derivatives suggests that both the C-4 and N-1 substitutions are important in retaining cytotoxicity against cancer cells. In the present study, we are interested in exploring the effect of additional C-4 and N-1 substitution diversity on possible gains of anticancer activity in the resulting analogs. To this end, we have designed, synthesized, and evaluated a novel series of pyrazolo[3,4-d]pyrimidine derivatives where the C-4 moiety ranges from chlorine to unsubstituted and substituted hydrazines, and the N-1 substitution is either an ethyl or a benzyl group (Scheme 1).

## 2. Results and Discussion

### 2.1. Chemistry

From commercially available allopurinol, compounds **2**, **3**, **4**, **5a**–**5g**, **6**, **7** and **8a**–**8c** were efficiently synthesized according to the synthetic route outlined in Scheme 1. Specifically, 4-chloro-1*H*-pyrazolo[3,4-d]pyrimidine (**2**) was obtained from the chlorination of allopurinol with POCl_3_. [22] *N*-Ethylation and benzylation of **2** with bromoethane or benzyl bromide under alkali conditions gave 4-chloro-1-ethyl-1*H*-pyrazolo[3,4-d]pyrimidine (**3**) and 1-benzyl-4-chloro-1*H*-pyrazolo[3,4-d]pyrimidine (**6**), respectively. The reaction of **3** with NH_2_NH_2_ affords 1-ethyl-4-hydrazinyl-1*H*-pyrazolo[3,4-d]pyrimidine (**4**). Next, acylation reactions of **4** with 4-methylbenzene-1-sulfonyl chloride, 4-nitrobenzene-1-sulfonyl chloride, benzenesulfonyl chloride, 4-bromobenzene-1-sulfonyl chloride or acetylchloride, benzoyl chloride, and chloroacetyl chloride lead to compounds **5a**–**5g**, respectively. The mono-*N*-arylation of ethane-1, 2-diamine by **6** resulted in the formation of *N*1-(1-benzyl-1*H*-pyrazolo[3,4-d]pyrimidin-4-yl)ethane-1,2-diamine (**7**). Finally, compounds **8a**–**8c** were formed by the acylation of **7** with benzenesulfonyl chloride, 4-nitrobenzene-1-sulfonyl chloride and 4-bromobenzene-1-sulfonyl chloride, respectively.

### 2.2. Biological Evaluation

#### 2.2.1. Cytotoxicity against Cancer Cell Lines

We first tested the cytotoxic activities of the newly synthesized allopurinol derivatives in the two human hepatoma carcinoma cell lines, BEL-7402 and SMMC-7221. For comparison, 17-AAG was included as a positive control in the assay. As shown in Table 1, compound **4** emerges as the only derivative having comparable activity to 17-AAG and **1** among all allopurinol derivatives with IC_50_ values of 25.5 and 35.2 μM against the 7402 and 7221 hepatoma cell lines, respectively. In comparison, 17-AAG inhibited BEL-7402 and SMMC-7221 cells with IC_50_ of 12.4 μM and 9.85 μM, respectively. Importantly, the discovery of compound **4** as the only derivative possessing toxicity towards cancer cells revealed that alkylating moiety on C-4, while important, is not essential to an active allopurinol derivative. As observed with compound **4**, when N-1 substitution is a simple ethyl group, a polar hydrazine on C-4 can bring about similar or greater cytotoxicity as an alkylating group in derivative **1**. However, such cytotoxicity of allopurinol derivatives is rather sensitive to C-4 substitution, with a relatively narrow window of opportunity for desirable activity.

When tested against another cancer cell line, MDA-MB-231, the allopurinol derivatives were found to exhibit moderate cytotoxicity. Shown in Table 2 are the relative survival rates of the analogs against MDA-MB-231 cells at 1 μM and 10 μM doses. For comparison, allopurinol, compound **1** and 17-AAG were included as references in the assay. Compound **4** reduced the survival rates of the cancer cells to 27.8% and 66.1% at 10 and 1 μM, again making it the most potent of all derivatives. While compound **4** is apparently more potent than allopurinol, it is less active than **1** and 17-AAG.

#### 2.2.2. Cytotoxicity towards MCF-10A Mammary Epithelial Cells

We next examined the cytotoxicity of the allopurinol derivatives in normal cells by treating the MCF-10A cells with compounds **4**, **5e**, and **5g** at various concentrations. Again, compound **1** and 17-AAG were used as references for comparison. As shown in Table 3, compared to compound **1**, whose activity is primarily attributable to its alkylating moiety, the new allopurinol derivatives exhibited significantly lower cytotoxicity than **1**. At 17.36 µM of IC_50_, the new analog **4** is much less toxic in normal cells than that of 17-AAG at 0.09 µM IC_50_. 

#### 2.2.3. XOD Inhibitory Activity

XOD catalyzes the conversion of xanthine to uric acid. Since allopurinol is a classic XOD inhibitor, it is of interest to evaluate the allopurinol derivatives for their XOD inhibitory activity as a result of structural modifications to gain on anticancer activity. The XOD inhibition assay revealed that all tested allopurinol derivatives have lost the distinct inhibitory activity towards XOD, in sharp contrast to the potency exhibited by allopurinol as a positive control. Thus, the anticancer activity of the allopurinol derivatives does not appear to be associated with XOD inhibitory activity. 

## 3. Materials and Methods

### 3.1. General Procedures for Chemical Synthesis

Allopurinol and xanthine oxidase were purchased from Sigma Aldrich (Shanghai, China). Dimethylsulfoxide (DMSO) was purchased from Amresco (Solon, OH, USA). All reagents were commercially available and were used without further purification unless otherwise indicated. Melting points were recorded with a micro melting point tester (Neware Technology Ltd., Guangdong, China) and are uncorrected. ^1^H and ^13^C-NMR spectra were obtained in DMSO-*d*_6_ or CDCl_3_ solutions on a Bruker AVII-400 spectrometer (Bruker, Fremont, CA, USA) operating at 400 and 100 MHz, respectively. The chemical shift values are given in δ units with reference to internal (CD_3_)_4_Si, and signals are designated as s (singlet), br s (broad singlet), d (doublet), t (triplet), q (quadruplet), dd (doublet of doublets), and m (multiplet), with coupling constants given in Hertz (Hz). High-resolution mass spectra were obtained on a Waters Q-TOF-Premiter instrument (TOF-MS) (Micromass, Manchester, UK). Infrared (IR) spectra were obtained by Perkin-Elmer 16PC-FT infrared spectrometer (Perkin-Elmer, Waltham, MA, USA). Thin layer chromatography (TLC) was employed to routinely monitor the reaction samples and confirm the homogeneity of the analytical samples by using Kieselgel 60F254 (0.25 mm) silica gel TLC aluminium sheets (Xiya Reagent, Chengdu, Sichuan, China). Column chromatography was carried out using silica gel 60, 200–300 mesh (Qingdao Haiyang Chemical, Qingdao, China). 

#### 3.1.1. 4-Chloro-1*H*-pyrazolo[3,4-d]pyrimidine (**2**) 

A mixture of allopurinol (2.00 g, 14.69 mmol) and *N*,*N*-dimethylaniline (2.00 g, 16.52 mmol) was stirred in POCl_3_ (25 mL) at 80 °C for 2 h. The reaction mixture was diluted with water (35 mL), and extracted with ethyl acetate. The organic layer was washed with water and the organic phase was concentrated to dryness, and the residue was purified by column chromatography on silica gel using 4:1.5 petroleum ether/ethyl acetate as eluent to give **2** [22]: white flake solid, 70 % yield.

#### 3.1.2. 4-Chloro-1-ethyl-1*H*-pyrazolo[3,4-d]pyrimidine (**3**)

To a solution of 4-chloro-1*H*-pyrazolo[3,4-d]pyrimidine 2 (0.1 g, 0.65 mmol) in anhydrous DMF (dimethylformamide, 5 mL ), TEA (trimethylamine, 0.20 g, 1.95 mmol) was firstly added, and mixture was stirred at room temperature for 30 min. Then, bromoethane (0.084 g, 0.78 mmol) was added and the mixture was stirred at room temperature for 1 h, and KI was then added. Then, the reaction mixture was diluted with water (15 mL), acidified with HCl and extracted with ethyl acetate. At last, the organic layer was washed with water and the organic phase was concentrated to dryness, and the residue was purified by column chromatography on silica gel using 10:1 petroleum ether/ethyl acetate as eluent to give 3: white powder, 76% yield, mp 76–77 °C; ^1^H-NMR (400 MHz, DMSO-*d*_6_) δ: 8.87 (s, 1H, CH), 8.49 (s, 1H, CH), 4.51 (q, *J* = 7.24 Hz, 2H, CH_2_), 1.45(t, *J* = 7.26 Hz, 3H, CH_3_); ^13^C-NMR (100 MHz, DMSO-*d*_6_) 156.7, 150.8, 150.4, 134.4, 106.1, 42.4, 15.1; IR (KBr, ν, cm^−1^): 3466, 3094, 2924, 2854, 1690, 1575, 1538, 1460, 1389, 1283, 1209, 1131, 1007, 956, 782, 680, 594, 533, 406. HRMS (ESI) calcd. for C_7_H_8_ClN_4_: [M + H]^+^ 183.0437, found 183.0535.

#### 3.1.3. 1-Ethyl-4-hydrazinyl-1*H*-pyrazolo[3,4-d]pyrimidine (**4**)

To a solution of 4-chloro-1-ethyl-1*H*-pyrazolo[3,4-d]pyrimidine 3 (0.1 g, 0.56 mmol) in acetonitrile (5 mL), hydrazine hydrate(0.034 g, 0.68 mmol) was firstly added. Then, the mixture was stirred at room temperature for 30 min. Finally, the reaction mixture was concentrated to dryness, and the residue was purified by recrystallization using ethyl acetate to give **4**: 75 % yield, mp 198–200 °C; ^1^H-NMR (400 MHz, DMSO-*d*_6_) δ: 8.29 (s, 1H, CH), 8.05 (s, 1H, CH), 4.85 (s, 2H, NH_2_), 4.65 (s, 1H, NH), 4.30 (q, *J* = 7.18 Hz, 2H, CH_2_), 1.36 (t, *J* = 7.22 Hz, 3H, CH_3_); ^13^C-NMR (100 MHz, DMSO-*d*_6_) 161.0, 155.4, 153.7, 135.2, 99.1, 41.5, 15.2; IR (KBr, ν, cm^−1^): 1717, 1659, 1599, 1538, 1497, 1441, 1375, 1346, 1295, 1251, 1182, 1091, 962, 913, 860, 785, 694, 620, 541. HRMS (ESI) calcd. for C_7_H_11_N_6_: [M + H]^+^ 179.1045, found 179.1044.

#### 3.1.4. Synthesis of Compounds **5a**–**5g**

To a solution of 1-ethyl-4-hydrazinyl-1*H*-pyrazolo[3,4-d]pyrimidine **4** (0.3 g, 1.69 mmol) in THF (5 mL), pyridine (0.19 g, 2.41 mmol) was firstly added at 0 °C and the mixture was stirred at 0 °C for 1 h. Then, substituted benzenesulfonyl chloride (2.05 mmol) or different acyl chloride was added and the reaction continued for 1 h at 0 °C. The reaction mixture was then diluted with water (15 mL), and extracted with ethyl acetate. At last, the organic layer was washed with water and the organic phase was dried over MgSO_4_ and concentrated, and the residue was purified by column chromatography on silica gel using 2:1 petroleum ether/acetone as eluent to give **5a**–**5g**.

*N*′*-(1-Ethyl-1H-pyrazolo[3,4-d]pyrimidin-4-yl)-4-methylbenzenesulfonohydrazide* (**5a**): 61% yield, mp 186–187 °C; ^1^H-NMR (400 MHz, DMSO-*d*_6_) δ: 10.28 (br, 1H, NH), 10.04 (br, 1H, NH), 8.16 (s, 2H, CH), 7.73 (d, *J* = 8.20 Hz, 2H, ArH), 7.41 (d, *J* = 8.08 Hz, 2H, ArH), 4.34 (q, *J* = 7.18 Hz, 2H, CH_2_), 2.39 (s, 3H, CH_3_), 1.37 (t, *J* = 7.20 Hz, 3H, CH_3_); ^13^C-NMR (100 MHz, DMSO-*d*_6_) 161.2, 154.9, 153.2, 144.2, 135.8, 133.8, 130.0, 128.2, 99.5, 41.8, 21.5, 15.4; IR (KBr, ν, cm^−1^): 3437, 3194, 3052, 2924, 2855, 1923, 1592, 1499, 1454, 1336, 1292, 1245, 1162, 1091, 960, 917, 810, 789, 718, 658, 582, 538, 486, 423. HRMS (ESI) calcd. for C_14_H_17_N_6_O_2_S: [M + H]^+^ 333.1134, found 333.1128.

*N′-(1-Ethyl-1H-pyrazolo[3,4-d]pyrimidin-4-yl)-4-nitrobenzenesulfonohydrazide* (**5b**): 64% yield, mp 194–195 °C; ^1^H-NMR (400 MHz, DMSO-*d*_6_) δ: 10.78 (br, 1H, NH), 10.17 (br, 1H, NH), 8.43 (s, 2H, CH), 8.17 (d, *J* = 8.76 Hz, 2H, ArH), 8.09 (d, *J* = 8.76 Hz, 2H, ArH), 4.34 (q, *J* = 7.24 Hz, 2H, CH_2_), 1.37 (t, *J* = 7.24 Hz, 3H, CH_3_); ^13^C-NMR (100 MHz, DMSO-*d*_6_) 159.5, 157.9, 155.3, 152.6, 148.1, 138.5, 134.7, 129.6, 104.3, 46.6, 19.9; IR (KBr, ν, cm^−1^): 3364, 3262, 3106, 2625, 2855, 1698, 1657, 1597, 1530, 1449, 1350, 1312, 1242, 1171, 1088, 1010, 961, 855, 746, 703, 612, 462. HRMS (ESI) calcd. for C_13_H_14_N_7_O_4_S: [M + H]^+^ 364.0828, found 364.0837.

*N*′*-(1-Ethyl-1H-pyrazolo[3,4-d]pyrimidin-4-yl)benzenesulfonohydrazide* (**5c**): 65% yield, mp 194–195 °C; ^1^H-NMR (400 MHz, DMSO-*d*_6_) δ: 10.40 (br, 1H, NH), 10.10 (br, 1H, NH), 8.16 (s, 2H, CH), 7.85 (d, *J* = 7.36 Hz, 2H, ArH), 7.60–7.69 (m, 3H, ArH), 4.33 (q, *J* = 7.12 Hz, 2H, CH_2_), 1.37 (t, *J* = 7.12 Hz, 3H, CH_3_); ^13^C-NMR (100 MHz, DMSO-*d*_6_) 160.4, 154.9, 153.2, 138.8, 133.7, 129.6, 128.2, 99.6, 41.8, 15.2; IR (KBr, ν, cm^−1^): 3227, 3077, 2979, 2861, 1593, 1548, 1500, 1442, 1353, 1292, 1248, 1167, 1090, 1008, 959, 919, 857, 790, 739, 693, 637, 577, 542, 502. HRMS (ESI) calcd. for C_14_H_15_N_6_O_2_S: [M + H]^+^ 319.0977, found 319.0984.

*4-Bromo-N′-(1-ethyl-1H-pyrazolo[3,4-d]pyrimidin-4-yl)benzenesulfonohydrazide* (**5d**): 58% yield, mp 194–195 °C; ^1^H-NMR (400 MHz, DMSO-*d*_6_) δ: 10.49 (br, 1H, NH), 10.09 (br, 1H, NH), 8.16 (s, 2H, CH), 7.76–7.84 (m, 4H, ArH), 4.34 (q, *J* = 7.18 Hz, 2H, CH_2_), 1.38 (t, *J* = 7.20 Hz, 3H, CH_3_); ^13^C-NMR (100 MHz, DMSO-*d*_6_) 158.8, 154.9, 153.2, 137.7, 134.0, 132.7, 130.3, 127.8, 99.5, 41.8, 15.2; IR (KBr, ν, cm^−1^): 3361, 3259, 3185, 3130, 3090, 2924, 2854, 1913, 1658, 1598, 1565, 1537, 1388, 1318, 1282, 1159, 1087, 1066, 1007, 959, 907, 821, 759, 717, 674, 615, 575, 530. HRMS (ESI) calcd. for C_13_H_14_BrN_6_O_2_S: [M + H]^+^ 397.0082, found 397.0088.

*N′-(1-Ethyl-1H-pyrazolo[3,4-d]pyrimidin-4-yl)acetohydrazide* (**5e**): 54% yield, pale yellow oil, ^1^H-NMR (400 MHz, DMSO-*d*_6_) δ: 10.38 (br, 1H, NH), 10.09 (br, 1H, NH), 8.29 (s, 1H, CH), 8.16 (s, 1H, CH), 4.34 (q, *J* = 7.12 Hz, 2H, CH_2_), 2.03 (s, 3H, CH_3_), 1.37 (t, *J* = 7.20 Hz, 3H, CH_3_); ^13^C-NMR (100 MHz, DMSO-*d*_6_) 170.1, 160.5, 155.4, 153.5, 132.3, 99.1, 41.8, 21.1, 15.2; IR (KBr, ν, cm^−1^): 3200, 3056, 2925, 2855, 1956, 1634, 1592, 1449, 1350, 1277, 1230, 1156, 1074, 1012, 891, 762, 708, 663, 597, 532. HRMS (ESI) calcd. for C_9_H_13_N_6_O: [M + H]^+^ 221.1151, found 221.1148.

*N*′*-Benzoyl-N′-(1-ethyl-1H-pyrazolo[3,4-d]pyrimidin-4-yl)benzohydrazide* (**5f**): 45% yield, mp 144–146 °C; ^1^H-NMR (400 MHz, DMSO-d_6_) δ: 10.52 (br, 1H, NH), 8.67 (s, 1H, CH), 8.09 (s, 1H, CH), 7.92–7.96 (m, 4H, ArH), 7.80–7.82 (m, 2H, ArH), 7.74–7.76 (m, 2H, ArH), 7.68–7.70 (m, 2H, ArH), 4.49 (q, *J* = 7.30 Hz, 2H, CH_2_), 1.44 (t, *J* = 7.22 Hz, 3H, CH_3_); ^13^C-NMR (100 MHz, DMSO-*d*_6_) 171.7, 171.0, 166.3, 154.6, 153.7, 134.6, 133.7, 133.0, 132.4, 129.0, 128.9, 128.0, 127.9, 104.6, 42.4, 15.1; IR (KBr, ν, cm^−1^): 3261, 3057, 2925, 2854, 1714, 1664, 1631, 1578, 1552, 1527, 1486, 1454, 1421, 1341, 1313, 1270, 1105, 958, 913, 869, 821, 795, 708, 691, 578, 545. HRMS (ESI) calcd. for C_21_H_19_N_6_O_2_ :[M + H]^+^ 387.1569, found 387.1577.

*2-Chloro-N′-(1-ethyl-1H-pyrazolo[3,4-d]pyrimidin-4-yl)acetohydrazide* (**5g**): 49% yield, mp 202–204 °C; ^1^H-NMR (400 MHz, DMSO-*d*_6_) δ: 11.27 (br, 1H, NH), 10.28 (br, 1H, NH), 8.11–8.50 (m, 2H, CH), 4.37–4.41 (m, 4H, CH_2_), 1.41 (t, *J* = 7.20 Hz, 3H, CH_3_); ^13^C-NMR (100 MHz, DMSO-*d*_6_) 164.8, 150.4, 148.9, 146.3, 133.2, 96.4, 41.2, 40.1, 13.4; IR (KBr, ν, cm^−1^): 3428, 3134, 2949, 2629, 1722, 1663, 1611, 1499, 1403, 1345, 1223, 1106, 965, 884, 799, 700, 639, 527; HRMS (ESI) calcd. for C_9_H_12_ClN_6_O: [M + H]^+^ 255.0761, found 255.0794.

#### 3.1.5. 1-Benzyl-4-chloro-1*H*-pyrazolo[3,4-d]pyrimidine (**6**)

To a solution of 4-chloro-1*H*-pyrazolo[3,4-d]pyrimidine 2 (0.1 g, 0.65 mmol) in anhydrous DMF (5 mL ), TEA (0.22 g, 0.65 mmol) was firstly added and stirred at room temperature for 30 min. Then, a solution of benzyl bromide (0.11 g, 0.78 mmol) in anhydrous DMF (5 mL) was added and the mixture was reacted for 1 h, then KI was added and continue reaction. The reaction mixture was at last diluted with water (15 mL), and extracted with ethyl acetate. The organic layer was washed with water and the organic phase was dried over MgSO_4_ and concentrated, and the residue was purified by column chromatography on silica gel using 10:1 petroleum ether/ethyl acetate as eluent to give **6**: 65% yield, mp 62–63 °C; ^1^H-NMR (400 MHz, DMSO-*d*_6_) δ: 8.91 (s, 1H, CH), 8.53 (s, 1H, CH), 7.26–7.33 (m, 5H, ArH), 5.70 (s, 2H, CH_2_); ^13^C-NMR (100 MHz, DMSO-*d*_6_) 156.6, 151.3, 150.9, 137.2, 135.1, 129.0, 129.0, 128.1, 128.1, 128.0, 106.3, 50.8; IR (KBr, ν, cm^−1^): 3441, 3090, 2935, 1897, 1756, 1585, 1547, 1479, 1410, 1347, 1243, 1174, 1135, 950, 858, 783, 730, 698, 596, 534; HRMS (ESI) calcd. for C_12_H_10_ClN_4_: [M + H]^+^ 245.0594, found 245.0584.

#### 3.1.6. *N*1-(1-Benzyl-1*H/*-pyrazolo[3,4-d]pyrimidin-4-yl)ethane-1,2-diamine (**7**)

To a solution of 1-benzyl-4-chloro-1*H*-pyrazolo[3,4-d]pyrimidine 6 (0.10 g, 0.41 mmol) in acetonitrile (10 mL), ethane-1,2-diamine (0.025 g, 0.42 mmol) was firstly added and stirred at room temperature for 3 h. Then, the reaction mixture was concentrated to dryness, and the residue was purified by column chromatography on silica gel using 2:8 methylene chloride/methanol as eluent to give **7**: 56% yield, yellow oil, ^1^H-NMR (400 MHz, DMSO-*d*_6_) δ: 8.61 (br, 1H, NH), 8.25 (s, 1H, CH), 8.03 (s, 1H, CH), 7.19–7.32 (m, 5H, ArH), 5.50 (s, 2H, CH_2_), 3.59 (m, 2H, CH_2_), 3.13 (m, 2H, CH_2_), 2.88 (t, *J* = 6.10 Hz, 2H, NH_2_); ^13^C-NMR (100 MHz, DMSO-*d*_6_) 156.5, 155.8, 152.7, 137.3, 132.4, 128.5, 127.4, 100.5, 49.7, 41.3, 40.6; IR (KBr, ν, cm^−1^): 3275, 2936, 2104, 1618, 1571, 1495, 1454, 1352, 1293, 1182, 1117, 1042, 918, 790, 732, 699; HRMS (ESI) calcd. for C_14_H_17_N_6_: [M + H]^+^ 269.1515, found 269.1506.

#### 3.1.7. Synthesis of Compounds **8a**–**8c**

To a solution of 7 (0.32 g, 1.19 mmol) in DMF10 mL), TEA (0.18 g, 0.78 mmol) was firstly added at 0 °C and stirred for 10 min. Then, substituted benzenesulfonyl chloride (1.34 mmol) was added and stirred at 0 °C for 2 h. The reaction mixture was diluted with water (15 mL), and extracted with ethyl acetate. At last, the organic layer was washed with water and the organic phase was dried over MgSO_4_ and concentrated. Then, the residue was purified by column chromatography on silica gel using 2:2:6 petroleum ether/ethyl acetate/methanol as eluent to give **8a**–**8c**.

*N-(2-((1-Benzyl-1H-pyrazolo[3,4-d]pyrimidin-4-yl)amino)ethyl)benzenesulfonamide* (**8a**): 61% yield, mp 114–115 °C; ^1^H-NMR (400 MHz, DMSO-*d*_6_) δ: 8.35 (br d, *J* = 5.60 Hz, 1H, NH), 8.25 (s, 1H, CH), 8.08 (s, 1H, CH), 7.83 (t, *J* = 5.96 Hz, 1H, NH), 7.76–7.79 (m, 2H, ArH), 7.50–7.58 (m, 3H, ArH), 7.20–7.33 (m, 5H, ArH), 5.49 (s, 2H, CH_2_), 3.54 (m, 2H, CH_2_), 3.00 (m, 2H, CH_2_); ^13^C-NMR (100 MHz, DMSO-*d*_6_) 156.8, 156.2, 153.1, 140.7, 137.8, 132.8, 132.4, 129.6, 128.9, 128.0, 126.9, 101.0, 50.26, 42.2, 40.4; IR (KBr, ν, cm^−1^): 3539, 3400, 3325, 3148, 2925, 2862, 1614, 1572, 1537, 1492, 1450, 1380, 1305, 1153, 1087, 983, 903, 790, 727, 689, 647, 583; HRMS (ESI) calcd. for C_20_H_21_N_6_O_2_S: [M + H]^+^ 409.1447, found 409.1435.

*N-(2-((1-Benzyl-1H-pyrazolo[3,4-d]pyrimidin-4-yl)amino)ethyl)-4-nitrobenzenesulfonamide* (**8b**): 66% yield, mp 171–172 °C; ^1^H-NMR (400 MHz, DMSO-*d*_6_) δ: 8.30 (m, 3H), 8.21 (br, 1H, NH), 8.04 (s, 1H, CH), 7.99 (d, *J* = 8.72 Hz, 2H, ArH), 7.88 (br, 1H, NH), 7.23–7.31 (m, 3H, ArH), 7.19 (d, *J* = 7.24 Hz, 2H, ArH), 5.47 (s, 2H, CH_2_), 3.53 (m, 2H, CH_2_), 3.11 (m, 2H, CH_2_); ^13^C-NMR (100 MHz, DMSO-*d*_6_) 156.7, 156.2, 153.0, 149.7, 146.5, 137.7, 132.3, 128.9, 128.4, 127.9, 124.9, 100.9, 50.2, 42.1, 40.4; IR (KBr, ν, cm^−1^): 3405, 3232, 2925, 2854, 1611, 1570, 1528, 1494, 1437, 1378, 1352, 1345, 1313, 1274, 1252, 1169, 1067, 894, 855, 788, 739, 686, 651, 609, 559, 535, 462; HRMS (ESI) calcd. for C_20_H_20_N_7_O_4_S: [M + H]^+^ 454.1297, found 454.1292.

*N-(2-((1-benzyl-1H-pyrazolo[3,4-d]pyrimidin-4-yl)amino)ethyl)-4-bromobenzenesulfonamide* (**8c**): 64% yield, mp 136–137 °C; ^1^H-NMR (400 MHz, DMSO-*d*_6_) δ: 8.35 (br, 1H, NH), 8.25 (s, 1H, CH), 8.09 (s, 1H, CH), 7.94 (br, 1H, NH), 7.72 (m, 4H, ArH), 7.25–7.32 (m, 3H, ArH), 7.21 (d, *J* = 6.96 Hz, 2H, ArH), 5.50 (s, 2H, CH_2_), 3.54 (m, 2H, CH_2_), 3.03 (m, 2H, CH_2_); ^13^C-NMR (100 MHz, DMSO-*d*_6_) 156.3, 155.71, 152.6, 139.6, 137.3, 132.2, 131.9, 128.5, 127.5, 126.1, 100.5, 49.8, 41.6, 40.1; IR (KBr, ν, cm^−1^): 3374, 2924, 2854, 1729, 1663, 1591, 1460, 1409, 1255, 1120, 1043, 854; HRMS (ESI) calcd. For C_20_H_20_BrN_6_O_2_S: [M + H]^+^ 487.0552, found 487.0562.

### 3.2. Biological Assays 

#### 3.2.1. Cell Culture

MDA-MB-231 cell line was routinely cultured in phenol red-free DMEM (Dulbecco’s modified eagle medium) medium supplemented with 5% FBS, 4 mM glutamine, 1 mM sodium pyruvate, 100 IU/mL penicillin, 100 μg/mL streptomycin and 0.25 μg/mL amphotericin. Cultures were maintained in 5% carbon dioxide at a temperature of 37 °C.

The Human Hepatoma Carcinoma Cell 7402 and 7221 cell lines were obtained from State Key of Biotherapy, Sichuan University, Sichuan, China. Logarithmically growing Human Hepatoma Carcinoma Cells 7402 and 7221 were incubated with 0.05% trypsin containing 1 mM EDTA at 37 °C for about 4 min until cells were non-adherent and formed a single cell suspension. Trypsin activity was neutralized by adding a 20-fold excess of serum-containing medium. Cells were cultured at 37 °C in an atmosphere of air and 5% CO_2_.

#### 3.2.2. Cell Survival/Growth Assay

For growth assay in the presence of 10^−5^ M and 10^−6^ M individual compound, MDA-MB-231 cells were plated in six-well plates at a density of 50,000 per well in DMEM medium supplemented with 5% FBS. The cells were then cultured for 5 days, while equal treatment volumes of DMSO were used as the vehicle control. Cell numbers were counted with a Coulter instrument (Beckman-Coulter, Brea, CA, USA). The ratio of compound treated cell numbers to vehicle treated cell numbers was defined as survival ratio.

Cell viability of human hepatoma carcinoma cell 7402 and 7221 were assayed by 3-(4,5-dimethylthiazol-2-yl)-2,5-diphenyltetrazolium bromide (MTT). Briefly, human hepatoma carcinoma cell 7402 and 7221 were plated at a density of 1 × 10^5^ cells/mL into 96-well plates and 7 × 10^3^ cells/well plates, respectively. After overnight growth, cells were pretreated with a series of concentrations of acacetin for 24 h. The final concentrations of dimethyl sulfoxide in the culture medium were <0.1%. At the end of treatment, 10 μL of MTT was added, and cells were incubated for a further 4 h. Cell viability was determined by scanning with an ELISA reader with a 570 nm filter (BioTek, Winooski, VE, USA).

MCF-10A cells were purchased from ATCC (Manassas, VA, USA) and were maintained in DMEM/F12 medium containing 1.05 mM CaCl_2_, 100 mg/mL cholera toxin, 5% horse serum, 10 μg/mL insulin, 500 ng/mL hydrocortisone and 1% penicillin-streptomycin. The cells were cultured in cell culture incubators, which were set to temperatures of 37 °C and supplemented with 5% CO_2_. MCF-10A cells were plated at a density of 3 × 10^3^ cells per well in 96-well plates, incubated overnight and then treated with serial dilutions of each individual drug using doses that closely corresponded to the individual IC_50_ values. After 72 h of exposure, the viable cell growth was measured using the Cell Titer-Glo luminescent cell viability assay kit (Promega, Madison, WI, USA).

#### 3.2.3. Determination of Xanthine Oxidase (XOD) Inhibitory Activity

Measurement of XOD activity was carried out according to the reference method with slight modifications [23]. First, 1092 μL of 0.1 unit of XOD in buffer (200 mM sodium pyrophosphate/HCl, pH 7.5) and 2 μL (5, 10, 15, 25 μM) of the test extracts or compounds in DMSO were mixed at 37 °C for 5 min. The control group did not contain a test agent. The reaction was started by adding 200 μL of 0.6 mM xanthine in doubly distilled water to the mixture. The reaction mixture was incubated at ambient temperature. Finally, the absorption increments at 295 nm indicating the formation of uric acid were determined every minute up to 8 min. Allopurinol was used as a positive control. Three replicates were made for each test sample. The percent inhibition ratio (%) was calculated according to the following equation:

% inhibition = [(rate of control reaction − rate of sample reaction)/rate of control reaction] × 100 (1)

## 4. Conclusions

A series of pyrazolo[3,4-d]pyrimidine derivatives from allopurinol were synthesized. *In vitro* cytotoxicity assays identified analog **4** as the most potent compound against breast cancer MDA-MB-231 and human hepatoma carcinoma BEL-7402 and SMMC-7221 cell lines. Its IC_50_ values of 25.5 and 35.2 μM are comparable to those of 17-AAG against BEL-7402 and SMMC-7221 cell lines, respectively. Importantly, the discovery of compound **4** with much greater potency than **5g** sheds light on another venue of structural variation that can achieve cytotoxicity against cancer cells without the alkylating moiety. This finding thus provides strong support to the conclusion that the alkylating moiety in pyrazolo[3,4-d]pyrimidine derivatives of allopurinol, while important, is not essential to conferring anticancer activity. Compared with 17-AAG, the much lower cytotoxicity of **4** toward normal cells make it a promising lead compound for further development toward anticancer agents of desirable pharmaceutical profiles. Additional studies on anticancer mechanisms of **4** are under way in our laboratories. 

## Figures and Tables

**Table 1:** *In vitro* activities of allopurinol derivatives against human hepatoma carcinoma 7402 and 7221 cell lines.

Compounds	IC_50_(μM) ^a^	
	BEL-7402	SMMC-7221
17-AAG	12.4 ± 0.1	9.85 ± 0.08
**1**	18.7 [19]	9.03 [19]
**3**	>100 ^b^	>100 ^b^
**4**	25.5 ± 0.4	35.2 ± 0.4
**5a**	>100 ^b^	>100 ^b^
**5b**	>100 ^b^	>100 ^b^
**5c**	>100 ^b^	>100 ^b^
**5d**	>100 ^b^	>100 ^b^
**5e**	>100 ^b^	>100 ^b^
**5f**	>100 ^b^	>100 ^b^
**5g**	>100 ^b^	>100 ^b^
**6**	>100 ^b^	>100 ^b^
**7**	>100 ^b^	>100 ^b^
**8a**	66.61 ± 0.62	>100 ^b^
**8b**	>100 ^b^	81.46 ± 0.78
**8c**	>100 ^b^	>100 ^b^

17-AAG: Tanespimycin. ^a^ IC_50_ value represents the drug concentration required to inhibit cancer cell replication by 50%. ^b^ The compounds were tested up to a concentration of 100 μM.

**Table 2:** *In vitro* activity of allopurinol derivatives against MDA-MB-231 cell assays.

Compounds	Survival Rate (%)		Compounds	Survival Rate (%)	
	10 μM	1 μM		10 μM	1 μM
17-AAG	4.8 ± 0.5	6.7 ± 0.5	**5e**	61.3 ± 1.0	94.8 ± 0.5
allopurinol	51.7 ± 6.7	84.3 ± 7.7	**5f**	88.1 ± 1.0	102.5 ± 11.0
**1**	1.0 ± 0.6	40.2 ± 3.9	**5g**	105.4 ± 2.9	105.4 ± 4.8
**3**	67.0 ± 4.8	86.2 ± 8.6	**6**	75.7 ± 1.5	77.6 ± 9.1
**4**	27.8 ± 2.4	66.1 ± 0.5	**7**	57.5 ± 1.0	81.4 ± 7.2
**5a**	72.8 ± 3.9	89.1 ± 1.5	**8a**	58.4 ± 2.4	114..0 ± 9.1
**5b**	68.0 ± 2.4	92.9 ± 1.5	**8b**	71.8 ± 8.5	75.7 ± 2.4
**5c**	74.7 ± 0.1	75.7 ± 11.0	**8c**	78.5 ± 3.9	105.4 ± 8.6
**5d**	70.9 ± 1.0	80.5 ± 2.0	DMSO	98.7 ± 6.3	100.6 ± 1.4

DMSO: Dimethylsulfoxide

**Table 3:** Cytotoxicity towards MCF-10A.

Compounds	IC_50_ (μM)
**1**	5.59 ± 0.27
**4**	17.36 ± 0.69
**5e**	20.25 ± 0.81
**5g**	23.29 ± 0.71
17-AAG	0.09 ± 0.01

## Abbreviations

The following abbreviations are used in this manuscript:

XOD: Xanthine oxidase
17-AAG: 17-***N***-allylamino-17-demethoxy geldanamycin
Hsp90: Heat Shock Protein 90
TEA: Triethylamine
DMF: Dimethylformamide
DMEM: Dulbecco’s modified eagle medium
FBS: Fetal bovine Serum
MTT: 3-(4,5-dimethylthiazol-2-yl)-2,5-diphenyltetrazolium bromide
NIH: National Institute of Health
RCMI: Research Centers in Minority Institutions Program
NIMHD: National Institute on Minority Health and Health Disparities
